# Hypercalcaemia After a Sunny Holiday

**DOI:** 10.7759/cureus.108642

**Published:** 2026-05-11

**Authors:** Shannon Ferrie, Puneet Malhotra, Alan Amal Calist

**Affiliations:** 1 General Medicine, Whiston Hospital, Merseyside, GBR; 2 Respiratory Medicine, Whiston Hospital, Merseyside, GBR

**Keywords:** calcitriol, case report, granulomas, hypercalcaemia, sarcoidosis

## Abstract

Severe hypercalcaemia can be life-threatening, and presentation is often non-specific. We report a case of a man who returned from a sunny holiday in Portugal with abdominal symptoms, confusion, and severe hypercalcaemia with normal serum parathyroid hormone (PTH). Routine blood tests, including serum calcium, performed in primary care two months before his holiday, were normal. Imaging during admission revealed bilateral hilar lymphadenopathy. Endobronchial ultrasound-guided fine-needle aspiration (EBUS-FNA) from mediastinal lymph nodes showed multiple non-caseating granulomas, consistent with a diagnosis of sarcoidosis. Hypercalcaemia was attributed to excess calcitriol (1,25-dihydroxyvitamin D) production by activated macrophages within sarcoid granulomas, exacerbated by recent sunlight exposure. The patient responded to intravenous fluids and corticosteroids, with subsequent steroid-sparing treatment using methotrexate. This case highlights the importance of considering sarcoidosis in PTH-independent hypercalcaemia.

## Introduction

Hypercalcaemia is a potentially severe metabolic condition defined as a serum calcium concentration of ≥2.6mmol/L, after adjustment for albumin. The most common causes are primary hyperparathyroidism and malignancy, together accounting for approximately 90% of cases [1]. In the absence of elevated parathyroid hormone (PTH) or malignancy, alternative aetiologies should be considered, including granulomatous diseases such as sarcoidosis and tuberculosis.

Disorders of calcium metabolism are well recognised in sarcoidosis, with hypercalcaemia in 10% of patients and hypercalciuria in up to 60% [2]. This occurs due to increased levels of serum calcitriol (1,25-dihydroxyvitamin D), the biologically active form of vitamin D. Calcitriol increases intestinal calcium absorption, promotes bone resorption, and reduces renal calcium excretion [3]. Increased sunlight exposure may result in higher levels of 25-hydroxyviatmin D, which is subsequently converted to calcitriol, leading to hypercalcaemia [4].

We present a case of sarcoidosis-associated hypercalcaemia in a 53-year-old man presenting to the Emergency Department after a two-week sunny holiday to Portugal, emphasising an important non-PTH-mediated cause of hypercalcaemia and the impact of ultraviolet exposure.

## Case presentation

A 53-year-old man attended the Emergency Department feeling generally unwell, with constipation, nausea, and episodes of intermittent confusion after returning to the UK from a two-week holiday in Portugal. He had a past medical history of insulin-dependent type 2 diabetes mellitus and had never smoked. Two months prior to his holiday, he had seen his general practitioner (GP) for enlarged neck lymph nodes and had undergone a fine needle aspiration, which had shown “reactive changes”. Routine blood tests, including international normalised ratio (INR), glycated haemoglobin (HbA1c), and serum calcium at that time, were normal.

Physical examination on admission to hospital revealed clinical evidence of dehydration, mild splenomegaly, but normal vital signs and non-palpable lymph nodes. Electrocardiogram (ECG) showed normal sinus rhythm with a normal QTc interval. Chest X-ray demonstrated bilateral hilar lymphadenopathy.

Initial blood tests are shown in Table 1. 

**Table 1:** Admission blood tests with reference ranges eGFR: Estimated glomerular filtration rate; ALT: Alanine aminotransferase; ALP: Alkaline phosphatase; PTH: Parathyroid hormone

Blood Test	Value	Reference range
Full blood count	Within normal limits	N/A
Sodium	135 mmol/L	133-146 mmol/L
Potassium	4.6 mmol/L	3.5-5.3 mmol/L
Urea	12.8 mmol/L	2.5-7.8 mmol/L
Creatinine	203 µmol/L	65-104 µmol/L
eGFR	30 ml/min/1.73 m^2^	>60 ml/min/1.73 m^2^
Adjusted Calcium	3.71 mmol/L	2.2-2.6 mmol/L
Phosphate	0.98 mmol/L	0.8-1.5 mmol/L
Albumin	36 g/L	35-50 g/L
ALT	23 U/L	7-40 U/L
ALP	210 U/L	30-130 U/L
Bilirubin	11 µmol/L	0-20 µmol/L
PTH	<0.1 pmol/L	1.6-6.9 pmol/L

**Figure 1:**
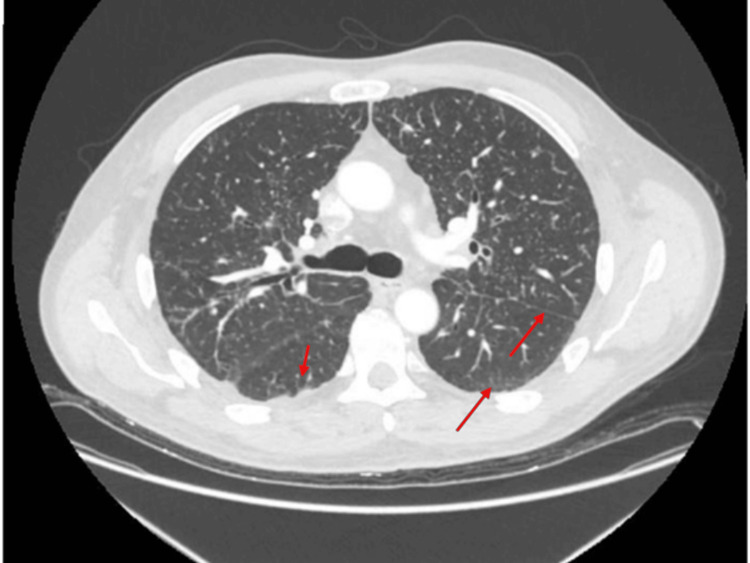
CT thorax (lung windows) demonstrating diffuse micro-nodules, interstitial thickening, and fissural beading. Arrows indicate the described abnormalities.

**Figure 2:**
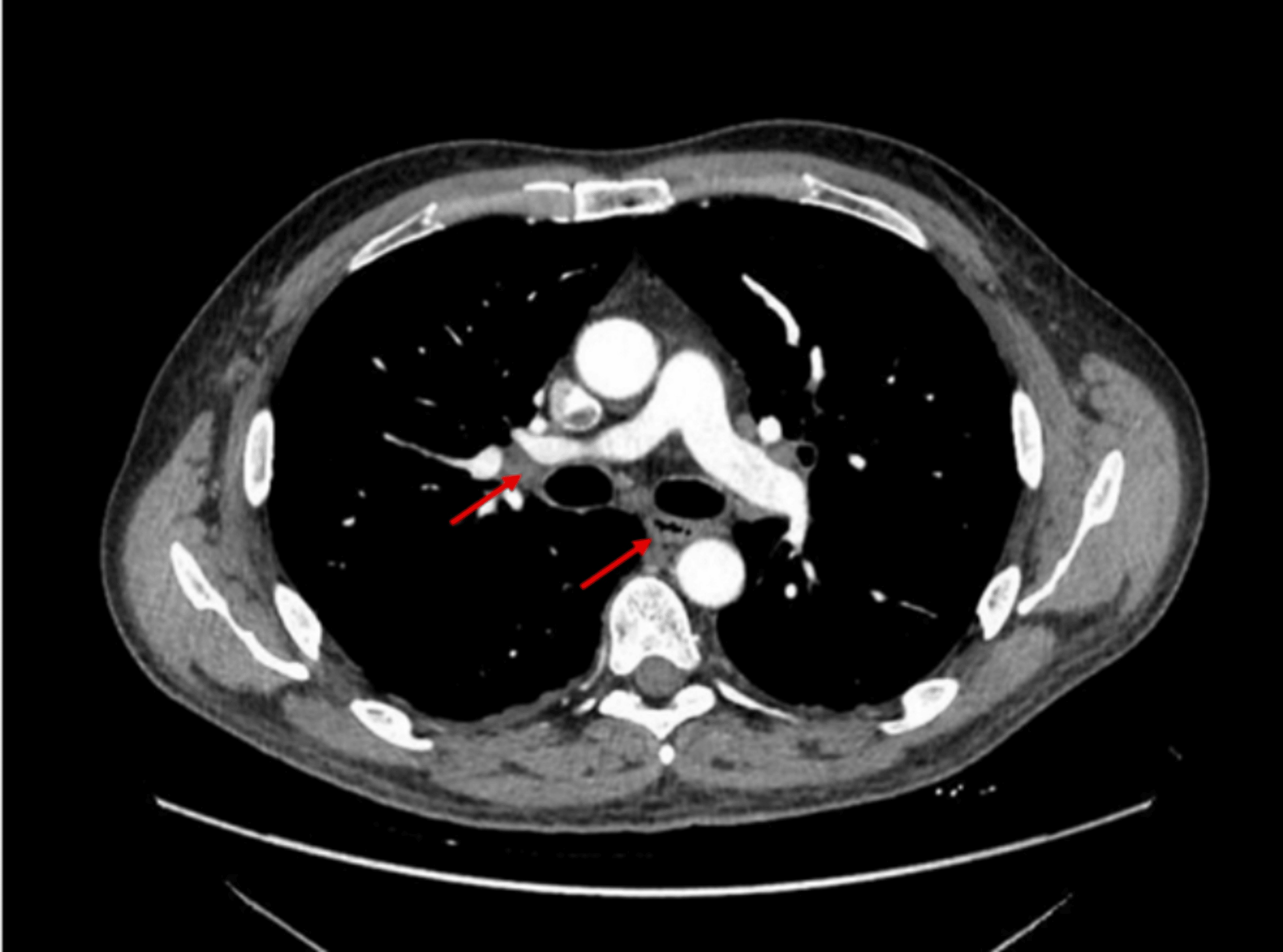
CT thorax, soft tissue windows showing mediastinal and right hilar lymphadenopathy. Arrows indicate the enlarged nodes.

He was given 2,000 ml of normal saline before having a CT thorax, abdomen, and pelvis, to assess for any evidence of occult malignancy. This demonstrated lung micro-nodularity, interstitial thickening, and fissural beading, with mediastinal and bilateral hilar lymph node enlargement (Figures 1, 2).

3,000 ml of normal saline was administered in the first 24 hours with close monitoring for fluid overload. An urgent endobronchial ultrasound-guided fine-needle aspiration (EBUS-FNA) of the mediastinal and hilar lymph nodes was performed, which, on cytological examination, showed multiple compact, non-caseating granulomata, consistent with the clinical and radiological picture of sarcoidosis. There were no extrapulmonary manifestations of sarcoidosis. Calcium levels were slow to decrease, so a stat dose of 8 mg dexamethasone was also given. He was then initiated on oral prednisolone and a proton pump inhibitor. His normal insulin dose was up-titrated to manage his blood glucose levels with the addition of steroids. 

Following management with IV fluids and oral prednisolone, his calcium levels normalised during the admission. He was discharged with a plan to continue oral steroids for 12-18 months and advised to wear SPF 50 sun cream in sunny environments. Disease response to steroids was good, with normal serum calcium on follow-up, and resolution of CT abnormalities. He did, however, suffer from weight gain and hyperglycaemia. He was, therefore, gradually weaned off steroids and started on methotrexate for disease control.

## Discussion

The diagnostic approach to hypercalcaemia relies on clinical assessment and targeted laboratory investigations to distinguish between primary hyperparathyroidism and malignancy, which together account for over 90% of cases [5]. In the remaining 10% of patients, alternative aetiologies must be systematically evaluated through thorough history-taking, physical examination, and appropriate investigations. A key initial step is differentiating PTH-mediated hypercalcaemia, such as primary hyperparathyroidism or familial hyperparathyroid syndromes, from non-PTH-mediated hypercalcaemia causes, including malignancy (e.g. myeloma), vitamin D intoxication, and granulomatous disease (e.g. sarcoidosis) [6].

In patients with suppressed serum PTH levels and no clear evidence of malignancy on initial clinical assessment, further biochemical evaluation is required. Measurement of 1,25-dihydroxyvitamin D and PTH-related peptide (PTHrP) levels can help identify vitamin D-mediated hypercalcaemia and malignancy, respectively. Cross-sectional imaging, such as computed tomography, is also warranted to assess for occult malignancy.

In patients presenting with hypercalcaemia in the absence of primary hyperparathyroidism or malignancy, sarcoidosis should be considered as a key differential diagnosis.

Severe hypercalcaemia, defined as a serum calcium concentration greater than 3.5 mmol/L, constitutes a medical emergency and requires prompt treatment irrespective of symptoms. There is a high risk of multi-organ dysfunction, including QT interval disruption and arrhythmias, acute kidney injury, and neurological dysfunction [7]. Initial management includes aggressive intravenous fluid resuscitation to correct intravascular volume depletion, which commonly accompanies severe hypercalcaemia and impairs renal calcium excretion [1]. Bisphosphonates are typically administered concurrently to inhibit osteoclast-mediated bone resorption. In this case, the markedly elevated calcium level necessitated rapid intravenous fluid replacement as part of acute management.

Calcium metabolism disorders are well recognised in sarcoidosis, with hypercalcaemia in 10% of patients and hypercalciuria in up to 60% [2]. The primary mechanism involves increased levels of calcitriol. Calcitriol increases intestinal calcium absorption, promotes bone resorption, and reduces renal calcium excretion, collectively resulting in elevated serum calcium concentrations.

**Figure 3:**
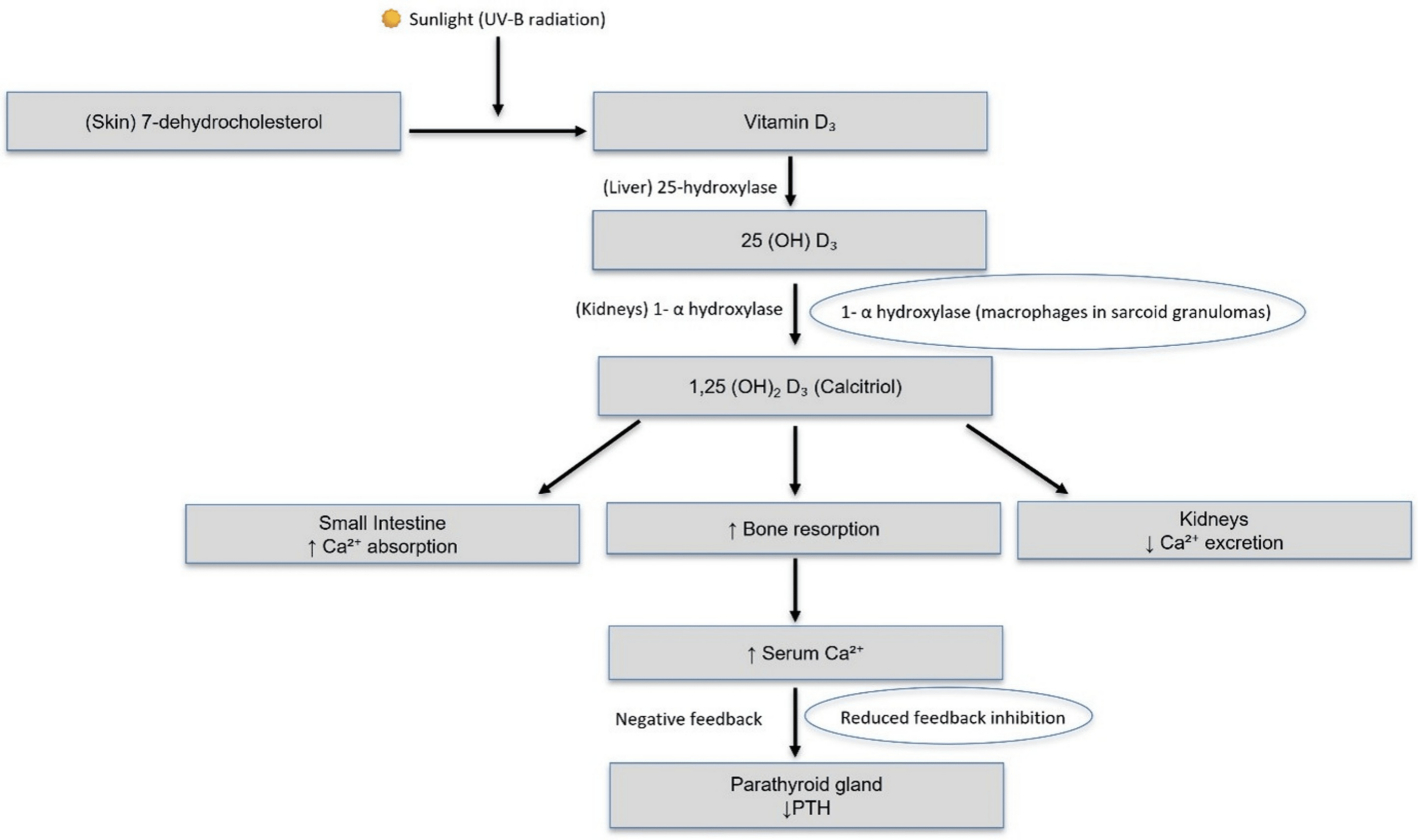
Calcium metabolism Flow chart demonstrating normal calcium and pathophysiology of hypercalcaemia in sarcoidosis. Higher levels of 1-alpha-hydoxylase result in higher levels of calcitriol and extrarenal calcium production. This production is not suppressed by normal negative feedback, thus is independent of PTH and normal calcium homeostasis. Created by the authors based on established physiology.

Under normal physiological conditions, conversion of 25-hydroxyvitamin D (25(OH)D) to calcitriol is tightly regulated by PTH and is appropriately suppressed in the presence of hypercalcaemia. However, in sarcoidosis, activated macrophages within granulomatous tissue, particularly in the lungs and lymph nodes, aberrantly express 1-alpha-hydroxylase, the enzyme responsible for this conversion [3,8-9]. This PTH-independent production of calcitriol is not subject to normal calcium-mediated negative feedback and therefore remains unsuppressed despite hypercalcaemia, resulting in persistent elevation of serum calcium (Figure 3).

Increased sunlight exposure raises circulating 25(OH)D levels, providing additional substrate for extrarenal 1-alpha-hydroxylase activity, thereby further increasing calcitriol production and exacerbating hypercalcaemia [4]. This mechanism may exacerbate hypercalcaemia in patients with sarcoidosis, as demonstrated in this case following prolonged sun exposure.

This case highlights the importance of considering sarcoidosis in the differential diagnosis of non-PTH-mediated hypercalcaemia and recognising environmental contributors such as increased sunlight exposure, which may exacerbate hypercalcaemia through extrarenal calcitriol production.

## Conclusions

Severe hypercalcaemia can be life-threatening, and presentation is often non-specific. The initial goal of the evaluation is to differentiate PTH-mediated hypercalcaemia from non-PTH mediated hypercalcaemia. In the context of suppressed PTH, sarcoidosis should be considered as an underlying cause. Hypercalcaemia is well-described in sarcoidosis and can present acutely as a first presentation to hospital. Sunlight exposure can precipitate hypercalcaemia in people with sarcoidosis by increasing the circulating levels of 25(OH)D.

## References

[1] Minisola S, Pepe J, Piemonte S, Cipriani C (2015). The diagnosis and management of hypercalcaemia. BMJ.

[2] Conron M, Young C, Beynon HL (2000). Calcium metabolism in sarcoidosis and its clinical implications. Rheumatology (Oxford).

[3] Papapoulos SE, Fraher LJ, Sandler LM, Clemens TL, Lewin IG, O’Riordan JL (1979). 1, 25-dihydroxycholecalciferol in the pathogenesis of the hypercalcaemia of sarcoidosis. Lancet.

[4] Taylor RL, Lynch HJ Jr, Wysor WG Jr (1963). Seasonal influence of sunlight on the hypercalcemia of sarcoidosis. Am J Med.

[5] Thillai M, Atkins CP, Crawshaw A (2025). BTS Clinical Statement on pulmonary sarcoidosis. Thorax.

[6] Burke RR, Rybicki BA, Rao DS (2010). Calcium and vitamin D in sarcoidosis: how to assess and manage. Semin Respir Crit Care Med.

[7] Carroll R, Matfin G (2010). Endocrine and metabolic emergencies: hypercalcaemia. Ther Adv Endocrinol Metab.

[8] Gwadera Ł, Białas AJ, Iwański MA, Górski P, Piotrowski WJ (2019). Sarcoidosis and calcium homeostasis disturbances - do we know where we stand?. Chron Respir Dis.

[9] Baughman RP, Valeyre D, Korsten P (2021). ERS clinical practice guidelines on treatment of sarcoidosis. Eur Respir J.

